# Tunable Dual-Mode Resonant Excitation of Dumbbell-Shaped Structures in the Mid-Infrared Band

**DOI:** 10.3390/nano15151181

**Published:** 2025-07-31

**Authors:** Tao Jiang, Yafei Li, Zhuangzhuang Xu, Xike Qian, Rui Shi, Xiufei Li, Meng Wang, Ze Li

**Affiliations:** 1 Research Center for Quantum Physics and Technologies, Inner Mongolia University, Hohhot 010021, China; taojiang@mail.imu.edu.cn (T.J.); xuzhuangzhuang@mail.imu.edu.cn (Z.X.); xike-qian@mail.imu.edu.cn (X.Q.); 0221121595@mail.imu.edu.cn (R.S.); xiufeili@imu.edu.cn (X.L.); 2 Key Laboratory of Semiconductor Photovoltaic Technology and Energy Materials of Inner Mongolia Autonomous Region, School of Physical Science and Technology, Inner Mongolia University, Hohhot 010021, China

**Keywords:** QBIC mode, electric anapole mode, metasurfaces, mid-infrared sensing

## Abstract

Metasurfaces have drawn extensive research attention for their unique optical properties and vast application potential. Among the various resonant modes induced in metasurfaces, BIC and electric anapole modes stand out as particularly interesting due to their distinctive physical characteristics. In this work, we designed and investigated novel dimeric dumbbell-shaped metasurfaces incorporating two independently tunable asymmetric parameters. This structural innovation enables the simultaneous excitation of both electric anapole and QBIC modes under normally incident MIR illumination. More importantly, by adjusting these two asymmetric parameters, one can independently tune the resonance peaks of the two modes, thereby overcoming the performance limits of conventional single-peak modulation. This metasurface design demonstrates outstanding performance for dielectric environment-sensing applications. We conducted a comprehensive investigation of the sensing sensitivity for dumbbell-shaped metasurfaces of various geometries. Our simulation results show that the circular-shaped configuration achieved high sensitivity, reaching 20,930 GHz/RIU. This work offers a novel design paradigm for multi-mode control and functionalization of metasurface structures.

## 1. Introduction

Metasurfaces are artificially engineered two-dimensional materials composed of subwavelength microstructure units [1,2,3]. In sensing applications, such metasurfaces leverage their subwavelength artificial microstructures to deliver unique advantages. By periodically arranging these resonant elements, one can achieve precise control over the phase, amplitude, polarization, and wavefront distribution of incident electromagnetic waves [4,5]. Metasurfaces can induce strong local field enhancement effects that amplify light–matter interactions [6,7]. When combined with their ultrathin form factor and exceptional design flexibility, this capability enables the optimization of sensing performance and the realization of highly miniaturized, integrated devices [8]. Metasurfaces can synergistically excite multiple modes, such as quasi-bound states in the continuum (QBIC) and electric anapole (EA) resonances, providing a versatile physical platform for high-degree-of-freedom spectral manipulation and multi-channel sensing integration [9,10].

Bound states in the continuum (BICs) are localized dark modes decoupled from the radiation continuum, featuring an infinite quality factor yet remaining difficult to observe experimentally [11,12]. By introducing symmetrical breaking, researchers can realize QBIC modes, which manifest as high-Q Fano resonance peaks in the spectrum [13,14]. EA modes suppress radiation through destructive far-field interference between electric and toroidal dipoles, resulting in highly localized near-field electromagnetic energy with low radiative loss and strong field enhancement [15,16]. QBIC and EA modes exhibit exceptional sensitivity and adaptability in sensing applications, enabling high-precision multiparameter detection and offering broad prospects for enhanced nonlinear optics, ultrasensitive sensing, and low-threshold lasing [17,18,19,20,21,22,23,24,25,26,27].

In recent years, research on the resonant properties of metasurface structures in the mid-infrared has advanced significantly [28,29]. Many metasurfaces now produce stable single high Q resonance peaks, offering excellent platforms for biomolecular sensing. Additionally, some structures use Fabry–Perot cavities to generate dual resonances of classical FP modes and QBIC, broadening applications in multi-gas detection in the mid-infrared [30,31,32,33]. This highlights the significant application potential of real-time monitoring of dielectric environment changes for biomolecular recognition, while the independent tuning of resonance peaks across multiple modes provides critical support for the development of multi-channel sensing systems [30,31,32,33,34,35,36,37,38,39]. However, in the current research, most metasurface structures can excite or manipulate only a single resonance mode across the broad mid-infrared spectrum, a limitation that severely constrains their potential for multi-channel sensing applications [40].

To address these challenges, this study introduces a metasurface architecture that breaks conventional symmetric constraints by incorporating two independently tunable asymmetric parameters. This dual-asymmetry design enables programmable coupling and modulation of two distinct resonance modes. Compared with single-mode systems, the proposed metasurfaces allow independent tuning of two resonance peaks across a broad spectral range by varying the asymmetric parameters. The resulting transmission spectra carry multidimensional optical information, significantly enhancing modal multiplexing capability. The spectral positions of both the QBIC and EA modes can be precisely controlled via the cooperative modulation of asymmetric parameters. Unlike common dielectric metasurfaces, the “hot spot” regions of the metallic structures in our model are located outside the structure, and this unique characteristic enables them to achieve higher sensitivity. The metasurface achieves a spectral shift sensitivity of 20,930 GHz/RIU for refractive index sensing. This translates variations in the refractive index of target molecules into quantifiable frequency shifts in the transmission spectrum. The proposed mechanism provides a physical foundation for multi-parameter sensing and establishes a dual-degree-of-freedom tuning paradigm, offering a novel strategy for multi-physics field sensing in the far-infrared regime.

## 2. Materials and Methods

**Figure 1:**
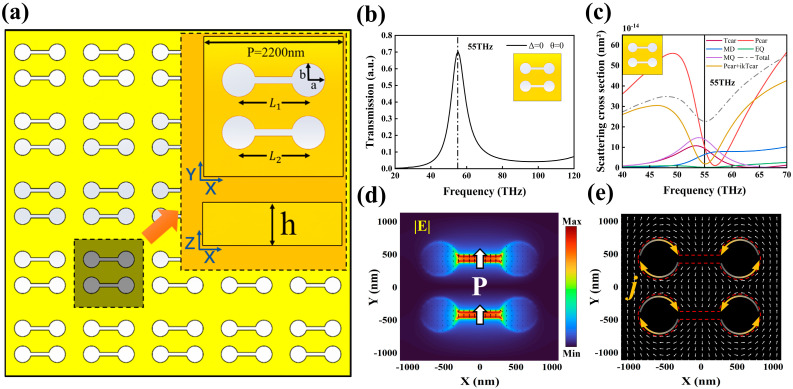
(**a**) Schematic diagram of the metasurfaces structure; (**b**) Transmission spectrum for Δ=0 and θ=0; (**c**) Mode decomposition image for Δ=0 and θ=0; (**d**) Electric field distribution and electric field lines in the XY plane; (**e**) Normalized current at the surface of the dumbbell-shaped structure.

The metasurface structure designed in this work, as shown in Figure 1a, is based on periodically arranged fundamental structural units with a periodic length of 2200 nm. The metasurface consists of a 700 nm-thick copper layer in which a hollowed-out region is implemented in the central area through an asymmetrical dimer-shaped dumbbell structure. The upper half of the dumbbell dimer comprises two spherical structures with a fixed center-to-center distance of $L_{1}=1100$ nm, while the width of the connecting rod structure in the middle is 110 nm. The center-to-center distance of the two spheres in the lower half is denoted as $L_{2}$, which can be adjusted according to design requirements, while the width of the middle connecting rod remains 110 nm. Additionally, the long and short axes of the spherical sections of the dumbbell structure are denoted as a and b, respectively, where the long axis a is fixed at 264 nm, while the short axis b is adjustable based on design needs.

In the structural design, we introduced two asymmetric parameters, Δ and θ, to characterize the geometric asymmetry. Specifically, the asymmetric parameter Δ is defined as follows:

(1) $$\Delta =\frac{L_{1}-L_{2}}{L_{1}}$$

where $L_{1}$ and $L_{2}$ represent the center-to-center distances between the spherical components of the upper and lower halves of the dumbbell dimer, respectively. The second asymmetry parameter θ is defined as follows:

(2) $$\theta =\frac{a-b}{a}$$

where a and b correspond to the long and short axes of the spherical sections of the dumbbell structure, respectively, with a and $L_{1}$ being fixed values.

To analyze the electromagnetic properties of the constructed structure, we employed the finite-difference time-domain (FDTD) method for numerical simulations. During these simulations, Bloch periodic boundary conditions were applied along the X and Y directions to model the electromagnetic behavior of an infinite periodic structure. Along the Z-direction at the top and bottom boundaries and in regions far from the metasurfaces, perfectly matched layer (PML) absorbing boundary conditions were imposed to effectively absorb outward-propagating electromagnetic waves, thereby simulating the electromagnetic field distribution in an open-space environment.

In the absence of introduced asymmetry, both asymmetry parameters are zero, indicating that the metasurface structure exhibits perfect mirror symmetry along both the X and Y axes. Introducing the asymmetry parameters Δ and θ partially disrupts this symmetry, thereby modulating the electromagnetic response characteristics of the structure. Figure 1b presents the transmission spectrum when both asymmetric parameters Δ and θ are set to zero. Under this condition, the transmission spectrum exhibits a resonance peak located at approximately 55 THz.

To further investigate the near-field contributions of various multipoles at the resonance peaks, including electric dipoles, toroidal dipoles, magnetic dipoles, electric quadrupoles, and magnetic quadrupoles, we systematically conducted a multipole expansion analysis of the respective moments. The different moments in the multipole expansion were computed from the calculated distribution of polarization current $J(r)=-i\omega P(r)=-i\omega \varepsilon_{0}(\varepsilon_{r}-1)E(r)$, where P is the polarization vector, ω is the angular frequency, $\varepsilon_{r}$ represents the relative permittivity of nanoparticle, and E(r) denotes the total electric field induced inside the particle. The multipole moments of the electric dipole (ED), magnetic dipole (MD), electric quadrupole (EQ), and magnetic quadrupole (MQ) can be derived as follows:

(3) $$ED:p_{\alpha }=-\frac{1}{i\omega }\left\{\int d^{3}rJ_{\alpha }^{\omega }j_{0}\left(kr\right)+\frac{k^{2}}{2}\int d^{3}r\left[3\left(r\cdot J_{\omega }\right)r_{\alpha }-r^{2}J_{\alpha }^{\omega }\right]\frac{j_{2\left(kr\right)}}{{\left(kr\right)}^{2}}\right\},$$

(4) $$MD:m_{\alpha }=\frac{3}{2}\int d^{3}r{\left(r\times J_{\omega }\right)}_{\alpha }\frac{j_{1}\left(kr\right)}{kr},$$

$$EQ:Q_{\alpha \beta }^{e}=-\frac{3}{i\omega }\left\{\begin{array}{c} \int d^{3}r\left[3\left(r_{\beta }J_{\alpha }^{\omega }+r_{\alpha }J_{\beta }^{\omega }\right)-2\left(r\cdot J_{\omega }\right)\delta_{\alpha \beta }\right]\frac{j_{1}(kr)}{kr} \end{array}\right.$$

(5) $$\left.+2k^{2}\int d^{3}r\left[5r_{\alpha }r_{\beta }\left(r\cdot J_{\omega }\right)-\left(r_{\alpha }J_{\beta }+r_{\alpha }J_{\beta }\right)r^{2}-r^{2}\left(r\cdot J_{\omega }\right)\delta_{\alpha \beta }\right]\frac{j_{3\left(kr\right)}}{{\left(kr\right)}^{3}}\right\},$$

(6) $$MQ:Q_{\alpha \beta }^{m}=15\int d^{3}r\left\{r_{\alpha }{\left(r\times J_{\omega }\right)}_{\beta }+r_{\beta }{\left(r\times J_{\omega }\right)}_{\alpha }\right\}\frac{j_{2}\left(kr\right)}{{\left(kr\right)}^{2}},$$

where k is wavenumber, $\alpha ,\beta =x,y,z,p_{\alpha }$,$m_{\alpha }$,$Q_{\alpha \beta }^{e}$, and $Q_{\alpha \beta }^{m}$ are the spherical multipole moments expressed in the Cartesian coordinates, and $j_{0,1,2}$ is the spherical Bessel function of the zeroth, first, and second order.

Next, the relative radiated powers to the far-field radiation from each multipole are as follows:

(7) $$I^{p}=\frac{2\omega^{4}}{3c^{3}}{\left|p_{\alpha }\right|}^{2},$$

(8) $$I^{m}=\frac{2\omega^{4}}{3c^{3}}{\left|m_{\alpha }\right|}^{2},$$

(9) $$I^{Qe}=\frac{\omega^{6}}{5c^{5}}{\left|Q_{\alpha \beta }^{e}\right|}^{2},$$

(10) $$I^{Qm}=\frac{\omega^{6}}{20c^{5}}{\left|Q_{\alpha \beta }^{m}\right|}^{2},$$

(11) $$I^{total}=I^{p}+I^{m}+I^{Qe}+I^{Qm}.$$

To validate the effective excitation of the toroidal dipole and EA mode in the periodic system, we calculate the scattering efficiencies of the Cartesian electric dipole ($p_{Car}$) and electric toroidal dipole ($T_{Car}$) in the Cartesian coordinate system. Electric toroidal dipole moments emerge as higher-order terms in the expansion of electric dipole moments: $ED=p_{Car}+ikT_{Car}$, specifically derived by applying the small-argument approximation to spherical Bessel functions: $j_{0}\left(k_{0}r\right)\approx 1-(k_{0}r{)}^{2}/6$, $j_{1}\left(k_{0}r\right)\approx k_{0}r/3$, $j_{2}\left(k_{0}r\right)\approx (k_{0}r{)}^{2}/15$. So, the multipole moments and the relative radiated powers of $p_{Car}$ and $T_{Car}$ can be expressed as follows [41,42,43,44,45,46,47,48,49]:

(12) $$p_{Car}=\frac{1}{-i\omega }\int d^{3}rJ\left(r\right),$$

(13) $$T_{Car}=\frac{1}{10c}\int d^{3}r\left[\left(r\cdot J\left(r\right)\right)r-2r^{2}J\left(r\right)\right],$$

(14) $$I^{p_{car}}=\frac{2\omega^{4}}{3c^{3}}{\left|p_{car}\right|}^{2},$$

(15) $$I^{T_{car}}=\frac{2\omega^{6}}{3c^{5}}{\left|T_{car}\right|}^{2}.$$

Figure 1c further illustrates the spectral mode decomposition of the structure in the vicinity of the resonance peak. Analyzing the modes within this frequency range, it can be observed that the resonance peak near 55 THz arises from the interference and coupling effects between the electric dipole mode and the toroidal dipole mode. Based on this analysis, it can be concluded that the resonance peak in this frequency range corresponds to an EA mode resonance, which is a localized resonance phenomenon induced by the interaction between the electric dipole and toroidal dipole modes.

Figure 1d shows the electric field distribution and electric field lines at the resonant frequency, indicating that a “hot spot” region forms in the middle of the perforated area at this frequency, where an electric dipole along the *Y*-axis is generated. According to Figure 1e, when incident light irradiates the metasurface, circular currents are induced in the circular perforated regions on the structure’s surface. The currents induced in the two spheres of the dumbbell structure flow in opposite directions, thereby exciting a toroidal dipole mode. In the far-field region, interference occurs between the toroidal dipole and the electric dipole, ultimately leading to their mutual cancellation and the formation of an EA mode.

**Figure 2:**
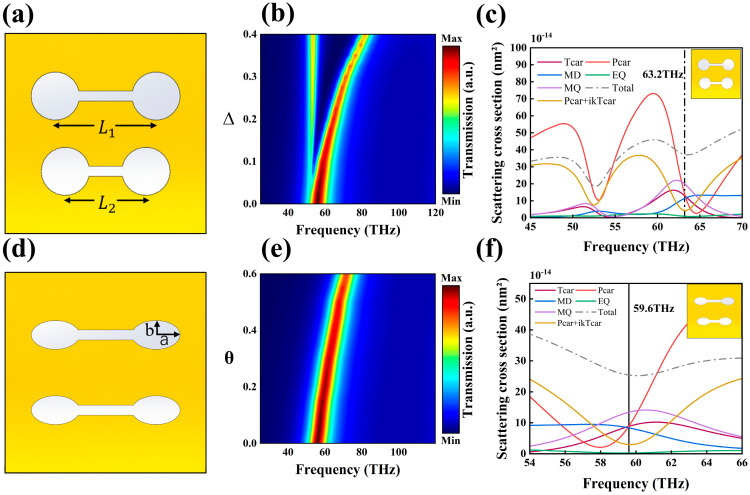
(**a**) Schematic diagram of the structure for Δ=0.2, θ=0; (**b**) Heatmap of parameter sweep for Δ with θ fixed; (**c**) Eigenmode decomposition for Δ=0.2, θ=0; (**d**) Schematic diagram of the structure for Δ=0, θ=0.4; (**e**) Heatmap of parameter sweep for θ with Δ fixed; (**f**) Eigenmode decomposition for Δ=0, θ=0.4.

Figure 2a,d illustrate the geometric configurations of the unit cell structure under different asymmetry parameter combinations, specifically (Δ=0.2, θ=0) and (Δ=0, θ=0.4), respectively. Mode decomposition analysis, as shown in Figure 2b,e, reveals that when the asymmetric parameter Δ is introduced (Δ=0.2), the initially singular EA mode undergoes splitting due to symmetrical breaking, resulting in the formation of a dual-resonance peak structure. The high-frequency peak corresponds to the classical EA mode, whose radiation-suppression mechanism arises from the destructive interference between the electric dipole and toroidal dipole. In contrast, the low-frequency peak exhibits characteristics of QBIC mode. In the system where only the parameter Δ is varied (Figure 2b), the resonance wavelength of the QBIC mode remains highly stable as Δ increases, whereas the EA mode exhibits pronounced blue shift. Moreover, the magnitude of this shift is positively correlated with Δ. This indicates that the introduction of a single parameter Δ enables precise tuning of the EA mode while having a negligible impact on the resonance position of the QBIC mode. In Supplementary Information S1, we also investigate the influence of the asymmetry parameter Δ on the Q values of the two modes. For the QBIC mode, the Q factor is inversely proportional to the square of the asymmetry parameter Δ, which clearly indicates that this mode is a symmetry-protected QBIC resonance.

Further analysis of the tuning effect of the single parameter θ (Figure 2e) revealed that the introduction of θ induced a blue shift in the resonance peak of the EA mode, with the shift magnitude positively correlated with θ. However, no QBIC mode was observed in this case. It is worth noting that the influence of θ is not solely limited to the EA mode; rather, its fundamental impact may involve broader mode coupling interactions. The absence of the QBIC mode in the present simulation is attributed to the lack of an introduced Δ parameter, indicating that the formation of the QBIC mode requires specific symmetry-breaking conditions triggered by Δ. To elucidate the potential role of the parameter θ in the excitation of the QBIC mode, further study implemented dual parameter tuning (Δ, θ). By conducting a parameter space sweep, the synergistic effects of Δ and θ on the excitation threshold and radiation characteristics of the QBIC mode were systematically investigated.

**Figure 3:**
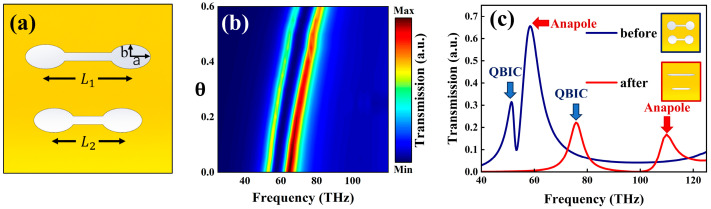
(**a**) Schematic diagram of the structure for Δ=0.2, θ=0.4; (**b**) Peak modulation response as a function of θ (with Δ fixed at 0.2); (**c**) Transmission contrast diagram of the asymmetric parameters Δ=0.05, θ=0 and Δ=0.45, θ=0.79.

As shown in Figure 3a, the simultaneous introduction of two asymmetric parameters (Δ and θ) into the metasurface enables independent manipulation of the resonant characteristics of both the QBIC and EA modes through coordinated parameter tuning. Simulation results indicate that, when Δ is fixed at 0.2 and θ is varied (as shown in the parameter evolution heatmap in Figure 3b), the resonance peaks of both modes exhibit similar sensitivity coefficients with respect to θ. This suggests that θ introduces an equivalent phase modulation effect on both modes by tuning the lattice anisotropy. In contrast, the primary role of Δ lies in modulating the radiation strength of the QBIC mode and the frequency separation between the two peaks. Notably, the resonance position of the QBIC mode remains relatively stable under variations in Δ, implying that its spectral location is primarily governed by θ. To evaluate the modulation range of the dual resonances, we selected two representative parameter sets with distinct structural asymmetries, (Δ=0.05, θ=0) and (Δ=0.45, θ=0.79), as shown in Figure 3c. The former serves as a reference case, since when Δ<0.05, the QBIC resonance becomes less pronounced. The latter set was chosen based on critical structural constraints; when Δ > 0.45, the EA resonance deteriorates and vanishes, and when θ > 0.79, the ellipse’s minor axis becomes smaller than the width of the central rod in the dumbbell-shaped structure. Simulations reveal that the QBIC resonance can be tuned over a spectral range from approximately 52 THz to 71 THz, while the EA mode spans a broader tuning range from 55 THz to 110 THz.

These findings highlight the distinct physical roles of the two asymmetric parameters: θ predominantly governs the global frequency shift of both modes by modifying the off-diagonal elements of the structure’s symmetry tensor. This results in a collective shift of the eigenmode band structure, synchronously affecting the spectral positions of the QBIC and EA modes. In contrast, Δ controls the degree of symmetry breaking, effectively activating the radiation leakage channel of the QBIC mode and enhancing intermodal coupling. As Δ increases, the coupling strength grows, leading to a monotonic expansion of the peak separation. This decoupled control of mode position (via θ) and mode coupling strength (via Δ) provides a new degree of freedom for metasurface engineering. It offers a versatile strategy for designing reconfigurable dual-mode resonators and multi-channel optical sensors. In integrated photonic multiplexing devices, θ can be used to define the operating wavelength of the QBIC mode, while Δ can be employed to tailor its coupling bandwidth with the EA mode, thereby enabling parallel detection across multiple physical channels.

We established a mathematical model to analyze the influence of the asymmetry parameters θ and Δ on the resonance positions of the QBIC and EA modes. The correlation between θ and the frequency shift of both resonance peaks was characterized by using a linear relationship model, whereas the tuning effect of Δ on the EA mode frequency shift was fitted using a quadratic polynomial model. The goodness-of-fit coefficients ($R^{2}$) for both models exceeded 0.98, confirming the validity of the proposed approach. The final quantitatively established relationship between the asymmetrical parameters and the resonance peak positions (in THz) is given as follows:

(16) $$f_{Anapole}=117.68\Delta^{1.7617}+57.347+26.37\theta$$

(17) $$f_{QBIC}=52.7+26.37\theta$$

By establishing a quantitative correlation model between the resonance peak shift and the two asymmetric parameters (θ, Δ), this study successfully achieves a coupled analysis of structural parameters and optical response characteristics. The mathematical model accurately characterizes the mapping relationship from the asymmetry parameter space to the resonance peak shift, with fitting residuals controlled within ±1 THz, significantly enhancing the predictive accuracy of the parameter sensitivity analysis. The inverse design approach developed within this theoretical framework provides a novel strategy for the development of micro-nano optical sensors with specific wavelength resolution capabilities.

In the field of advanced optical sensing technologies, electromagnetic resonances based on EA and QBIC modes have demonstrated significant advantages for biomolecular detection and monitoring of microenvironmental refractive index changes. EA modes enhance light–matter interaction efficiency, improving both the sensitivity and precision of sensors, while generating highly localized electromagnetic fields that facilitate the detection of low-concentration biomolecules or chemical species. QBIC modes, characterized by ultrahigh quality factors and extremely narrow resonance linewidths, enable high-precision detection. The combination of these two modes further enhances the sensor’s responsiveness to refractive index variations, achieving superior detection sensitivity. In this work, copper was employed due to its high electrical conductivity, effectively exciting surface plasmon resonances (SPR) and significantly enhancing sensing sensitivity through local field amplification. Moreover, copper can be readily fabricated into dumbbell-shaped nanostructures, where geometric parameter tuning enables the simultaneous support of multimode resonances, offering great potential for practical engineering applications.

Copper is well-suited for fabrication into complex micro-nano structures, such as dumbbell-shaped apertures. By tuning the geometric parameters of these structures, multi-modal resonance can be simultaneously supported, offering substantial engineering potential for advanced optical sensing applications.

In the numerical simulations conducted in this study, a polyvinyl alcohol (PVA) thin film was introduced on the substrate surface of the dual-dumbbell resonant structure as a dielectric support layer to accommodate the analyte solution under investigation. By systematically adjusting the refractive index of the surrounding medium, a pronounced spectral blue shift in the resonance frequency was observed. Based on the performance evaluation framework of optical biosensors, the sensing sensitivity S in this study is defined as the ratio of the resonance frequency shift to the variation in refractive index, as follows:

(18) $$S=\frac{\Delta f}{\Delta n}$$

This quantitative metric effectively characterizes the sensor’s responsiveness to variations in the external refractive index, where Δf represents the resonance frequency shift (measured in GHz) and Δn denotes the change in the surrounding refractive index (measured in refractive index units, RIU).

**Figure 4:**
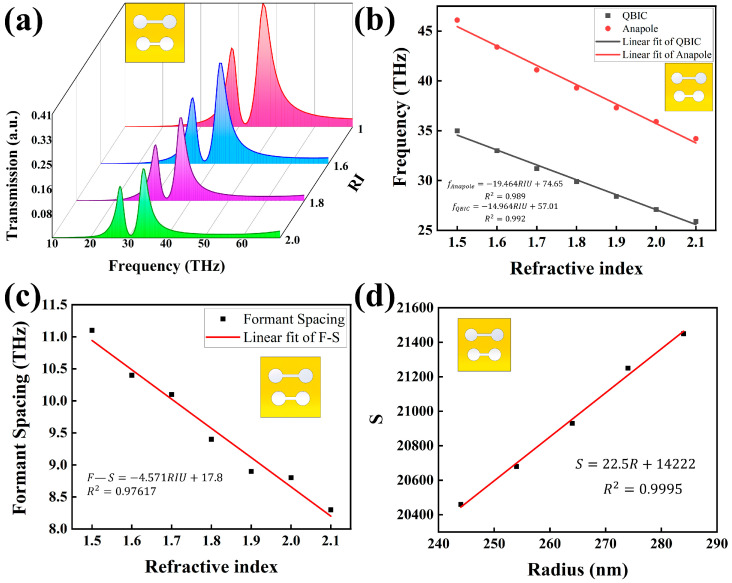
(**a**) Dual-resonance transmission spectra of the circular dumbbell-shaped metasurfaces with fixed asymmetry parameter Δ = 0.2 under varying ambient refractive indices. (**b**) Resonance frequency shifts of the QBIC and EA modes as a function of refractive index. (**c**) Formant spacing (FS) between the dual resonance peaks as a function of refractive index. (**d**) Refractive index sensitivity of circular dumbbell-shaped metasurfaces with varying resonator sizes under constant total footprint constraint.

Figure 4a presents the dual-resonance spectra of the metasurfaces with fixed asymmetry parameters (Δ=0.2, θ=0) under varying surrounding refractive indices. As the refractive index of the ambient medium increases, both the QBIC and EA resonances exhibit a distinct blueshift in frequency. Notably, the EA mode demonstrates a markedly higher sensitivity to refractive index variations compared to the QBIC mode. In particular, for refractive index changes ranging from 1.4 to 2.1, the EA-based resonance achieves a maximum sensitivity (S) of up to 20,930 GHz/RIU, highlighting its exceptional potential for detecting refractive index fluctuations in complex environments. As shown in Figure 4b, both resonance peaks display excellent linear dependence on refractive index changes within the range of 1.5 to 2.1, with regression coefficients ($R^{2}$) exceeding 0.98. This strong linearity confirms the robustness and accuracy of the proposed sensing model. Additionally, Figure 4c demonstrates that the spacing between the two resonance peaks—defined as formant spacing (FS)—exhibits a clear negative linear correlation with refractive index ($R^{2}>0.97$). This linear trend offers a complementary metric for refractive index quantification. The monotonic decrease in FS further indicates that the EA resonance is significantly more sensitive to refractive index perturbations than the QBIC mode, thereby emphasizing its superiority in high-precision sensing applications. Accordingly, the EA mode resonance was selected as the primary metric in subsequent sensitivity comparisons.

Figure 4d illustrates the refractive index sensitivity of the dual dumbbell metasurfaces as a function of the circular resonator radius. Numerical simulations revealed a positive correlation between the geometric parameter and the sensing performance-specifically, the sensitivity increasing significantly with the enlargement of the circular radius. Importantly, in the subsequent comparative studies involving multiple dual-dumbbell geometries, the total structural footprint was kept like eliminating confounding area-related effects. This area-constrained comparative approach ensures that the observed sensitivity differences are solely attributable to geometric shape variations, thereby enabling a more rigorous assessment of structural influence on sensing performance.

**Figure 5:**
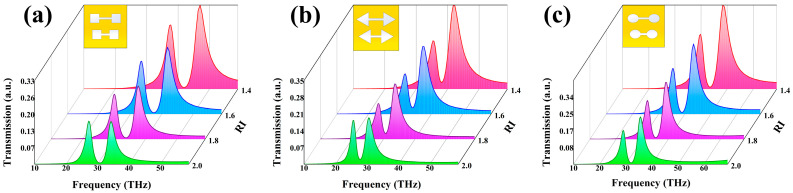
Sensitivity of dual-dumbbell structures with different geometries to varying refractive indices, (**a**) square, (**b**) triangular, (**c**) elliptical.

As illustrated in Figure 5, we investigated the sensitivity of double-dumbbell structures with identical footprint areas but different shapes to variations in the surrounding refractive index. Simulation results reveal that circular and elliptical structures exhibit higher sensitivity compared to square and triangular ones. We attribute this to the non-uniform field distribution at the edges of square and triangular geometries, which can lead to disrupted or dispersed current paths and consequently weaken the contribution of the toroidal dipole moment. Since the interference between the toroidal and electric dipole moments underpins the EA mode, this directly impacts the resonance sensitivity.

In practical metasurface fabrication, achieving perfectly circular double-dumbbell structures is often challenging. The study further demonstrates that elliptical metasurfaces also exhibit high sensitivity. Owing to their relatively low fabrication precision requirements, it is demonstrated that these metasurface structures can maintain strong sensing performance even when perfect circular geometries cannot be achieved during fabrication. This indicates that such metasurfaces are less dependent on stringent fabrication conditions, offering greater practical applicability and broader potential for real-world deployment.

## 3. Conclusions

This study presents a novel metasurface featuring a dumbbell-shaped configuration that enables the simultaneous excitation of QBIC and EA modes within a single structural unit in the mid-infrared regime. By innovatively introducing a dual asymmetry parameter modulation scheme, defined by Δ and θ, independent spectral tuning of the QBIC and EA resonances has been achieved for the first time. A quantitative relationship is established between the asymmetrical parameters and the corresponding resonance wavelengths, exhibiting a high degree of correlation ($R^{2}$ > 0.98). Furthermore, a systematic analysis of the sensing performance of dumbbell-shaped metasurfaces with various geometries confirms that circular structures offer superior sensing capabilities due to their optimized electromagnetic field distribution. This work provides a novel design paradigm for the development of micro- and nanoscale photonic devices with dual-channel spectral resolution.

## Data Availability

Data underlying the results presented in this article are not publicly available at this time but may be obtained from the authors upon reasonable request.

## Supplementary Materials

The following supporting information can be downloaded at: https://www.mdpi.com/article/10.3390/nano15151181/s1.
