# Supplementary material for: Tunable Dual-Mode Resonant Excitation of Dumbbell-Shaped Structures in the Mid-Infrared Band
Source: Nanomaterials (Basel). 2025 Jul 31;15(15):1181. doi: 10.3390/nano15151181 (PMC12348914; doi:10.3390/nano15151181)
Supplement: Supplementary file 1 [file nanomaterials-15-01181-s001.zip › nanomaterials-3726345-supplementary.pdf]

Supplementary Information for "Tunable dual-mode resonant excitation of dumbbell-shaped structures in the mid-infrared band"

Tao Jiang[1, ‡], Yafei Li[2, ‡], Zhuangzhuang Xu[1], Xike Qian[1], Rui Shi[1], Xiufei Li[1], Meng Wang[1,2,3 *] and Ze Li[1,2, *]

1.Research Center for Quantum Physics and Technologies, Inner Mongolia University, Hohhot, Inner Mongolia 010021, China.

2.Key Laboratory of Semiconductor Photovoltaic Technology and Energy Materials of Inner Mongolia Autonomous Region, School of Physical Science and Technology, Inner Mongolia University, Hohhot, Inner Mongolia 010021, China.

3.Institute of Information Photonics Technology and College of Applied Sciences, Beijing University of Technology, Beijing 100124, P. R. China

*wangmeng@imu.edu.cn *li@imu.edu.cn

S1: The Q values of the two modes under different asymmetry parameters Δ

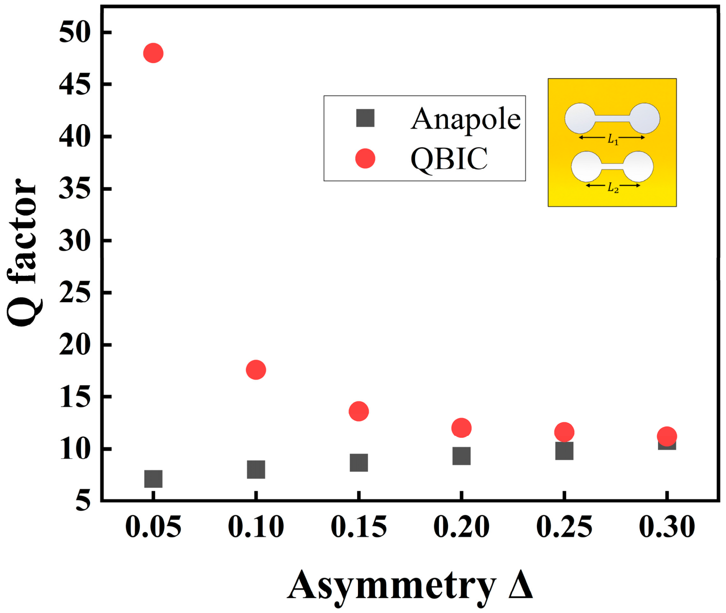

Fig.S1. The Q values of the two modes under different asymmetry parameters Δ

Our definition of the asymmetry parameter Δ is

$$\Delta = \frac{L_1 - L_2}{L_1}$$

In the work of many researchers on QBIC modes, it was found that the quality factor Q of the QBIC mode is positively correlated with the inverse square of the asymmetry parameter. We leak out the QBIC peak by varying L_2,In Figure S1, when an asymmetry parameter is

introduced into the structure, we found that the quality factor Q of the QBIC mode is positively correlated with the inverse square of the asymmetry parameter Δ, which corresponds to our results. Due to the material loss of copper, there is a certain deviation, but it still clearly indicates that this mode is a symmetry-protected QBIC resonance. In particular, for the Anapole mode, a slight enhancement was observed with the increase of Δ, and its quality factor Q has no positive correlation with the inverse square of the asymmetry parameter Δ. We believe this is another evidence to distinguish between the two modes.
